# A Direct Position-Determination Approach for Multiple Sources Based on Neural Network Computation

**DOI:** 10.3390/s18061925

**Published:** 2018-06-13

**Authors:** Xin Chen, Ding Wang, Jiexin Yin, Ying Wu

**Affiliations:** 1 National Digital Switching System Engineering and Technology Research Center, Zhengzhou 450002, China; ischen.xin@foxmail.com (X.C.); Cindyin0807@163.com (J.Y.); hnwuying22@163.com (Y.W.); 2 Zhengzhou Information Science and Technology Institute, Zhengzhou 450002, China

**Keywords:** direct position determination (DPD), neural network, multiple signal classification algorithm, maximum likelihood estimator

## Abstract

The most widely used localization technology is the two-step method that localizes transmitters by measuring one or more specified positioning parameters. Direct position determination (DPD) is a promising technique that directly localizes transmitters from sensor outputs and can offer superior localization performance. However, existing DPD algorithms such as maximum likelihood (ML)-based and multiple signal classification (MUSIC)-based estimations are computationally expensive, making it difficult to satisfy real-time demands. To solve this problem, we propose the use of a modular neural network for multiple-source DPD. In this method, the area of interest is divided into multiple sub-areas. Multilayer perceptron (MLP) neural networks are employed to detect the presence of a source in a sub-area and filter sources in other sub-areas, and radial basis function (RBF) neural networks are utilized for position estimation. Simulation results show that a number of appropriately trained neural networks can be successfully used for DPD. The performance of the proposed MLP-MLP-RBF method is comparable to the performance of the conventional MUSIC-based DPD algorithm for various signal-to-noise ratios and signal power ratios. Furthermore, the MLP-MLP-RBF network is less computationally intensive than the classical DPD algorithm and is therefore an attractive choice for real-time applications.

## 1. Introduction

In recent decades, neural network (NN) methods for localization have been studied intensively, with radial basis function (RBF) networks [1,2,3,4] and multilayer perceptron (MLP) networks [5,6,7,8] applied for localization. For real-time direction-of-arrival (DOA) estimation problems, a minimal resource allocation network (MRAN) for DOA estimation under array sensor failure in a noisy environment has been developed [9]. Results indicate the superior performance of MRAN-based DOA estimation in the presence of different antenna effects, failure conditions, and noise levels. A modular NN for the DOA estimation of two coherent sources has also been proposed [10]. A modified neural multiple-source tracking algorithm (MN-MUST) [11] was developed for real-time multiple-source tracking problems, wherein a spatial filtering stage was inserted to considerably improve the performance of the system. For wireless sensor networks (WSNs), a flexible MLP-based model for the accurate localization of sensors has been reported [12], with simulation experiments showing that the location accuracy can be enhanced by increasing the grid sensor density. A new artificial neural network (ANN) approach [13] has been developed to moderate the effect of miscellaneous noise sources and harsh factory conditions on the localization of wireless sensors. In terms of wireless communication systems, a novel ANN algorithm that utilizes time-of-arrival (TOA) and angle-of-arrival (AOA) measurements to locate mobile stations in non-line-of-sight environments has been proposed [14]. Additionally, a fingerprint-based localization methodology was applied in an experimental indoor environment, and the processing received signal strength indicator (RSSI) information for determining the position was used to train the NN [15]. Similarly, an MLP-NN has been applied to wireless local area networks [16], where a flexible mapping was built between the raw received signal strength (RSS) measurements and the position of the mobile terminal.

Clearly, the above localization methods are conventional two-step techniques that firstly estimate positioning parameters (such as AOA, TOA, and RSS) and then use these parameters to determine the source positions. It has been verified that conventional two-step localization methods are suboptimal, because these positioning parameters are estimated at each base station separately, without using the constraint that all parameters must correspond to the same transmitter. Weiss and Amar proposed the direct position determination (DPD) method [17,18,19,20,21], which directly localizes transmitters from sensor outputs. Compared with conventional two-step localization, DPD avoids the measurement-source association problem in the estimation of positioning parameters for multiple-source scenarios, leading to higher positioning accuracy. However, existing DPD methods, whether maximum likelihood (ML)-based algorithms [17] or multiple signal classification (MUSIC)-based algorithms [18], are computationally expensive, which makes it difficult to implement them in real time. To reduce the computation time without loss of localization performance, we present an NN method for DPD. To the best of our knowledge, no previous studies have applied the NN method to DPD. The difficulties in applying NNs to DPD include the high-dimensional input space caused by the large amount of raw data from multiple observers and the need for a large training set to cover different combinations of the multiple source locations, signal-to-noise ratios (SNRs), and signal power ratios (SPRs).

To solve the above problems, we propose a modular NN scheme for multiple-source DPD. The proposed method combines RBF and MLP NNs. In our scheme, the area of interest is divided into multiple sub-areas, and RBF-NNs are trained as source location estimators for each sub-area while MLP-NNs are trained to perform detection and filtering, which effectively reduces the size of the training set. The preprocessing operations of dimension reduction and normalization greatly reduce the dimension of the input space. Compared with conventional DPD methods such as ML-based and MUSIC-based algorithms, the main advantage of the proposed method is that it avoids the need for a peak search and the iteration process, which have high computational complexity, and can implement DPD almost instantaneously. In addition, the proposed method can achieve adaptive learning and has excellent generalization performance for samples outside the training set.

The remainder of this paper is organized as follows. The data model is described in Section 2. Section 3 introduces a modular NN with an MLP-MLP-RBF structure for DPD. The performance of the proposed method is studied and simulation results are presented in Section 4. Section 5 gives the conclusions and future work of the article. The main notation used in this paper is listed in Table 1.

## 2. Data Model

Our study aims at line-of-sight passive location scenario of ultra-short-wavelength signals, for the purpose of reconnaissance. Consider an outdoor scenario with J observer arrays, each consisting of $M_{j}$ sensors. Assume that there are P stationary transmitters radiating narrowband signals in the far field of the arrays and that the signals are uncorrelated. We presume $P<M_{j},j=1,2,\cdots ,J$ and that the signals received by the j-th array can be expressed as

(1) $$x_{j}(t)\;=\sum_{p=1}^{P}s_{jp}(t)a_{j}(u_{p})+n_{j}(t)$$

where $u_{p}$ is the position of the p-th source; $a_{j}(u_{p})$ is the steering vector of the p-th source for the j-th array; and $s_{jp}\left(t\right)$ denotes the complex envelope of the p-th source reaching the j-th array at time t, which has a circular complex Gaussian distribution $N^{C}(0,\sigma_{sp}^{2}),p=1,2,\cdots ,P$, where the variance $\sigma_{sp}^{2}$ is unknown. $n_{j}(t)$ is the background noise of the j-th array, which has a zero mean complex Gaussian distribution with covariance matrix $\sigma_{n}^{2}I$, where $\sigma_{n}^{2}$ is assumed to be known because it can be estimated or measured in practical applications [22]. The noise and signals are assumed to be mutually independent.

Equation (1) can be written in matrix form as

(2) $$x_{j}(t)=A_{j}s_{j}\left(t\right)+\;n_{j}(t)\;$$

where $A_{j}=[\;a_{j}(u_{1}),\;a_{j}(u_{2}),\cdots ,\;a_{j}(u_{P})]$ is the array manifold matrix of the j-th array, which has dimensions of $M_{j}\times P$, and $s_{j}(t)=[s_{j1}(t),s_{j2}(t),\cdots ,s_{jP}(t)]$.

The covariance matrix of the signals at the output of the j-th array is

(3) $$R_{j}=E\{x_{j}(t)\;x_{j}^{H}(t)\}=A_{j}P_{j}A_{j}^{H}+\sigma_{n}^{2}I$$

where $P_{j}=E\{s_{j}(t)s_{j}^{H}(t)\}$ is the covariance matrix of the signals’ complex envelope.

Assuming that the number of snapshots is K, the covariance matrix of the signals received by the j-th array can be estimated as

(4) $${\hat{R}}_{j}=\frac{1}{K}\sum_{k=1}^{K}x_{j}(k)x_{j}^{H}(k)$$

The SNR and SPR are defined as

(5) $$\mathrm{SNR}=10\log_{10}(\sigma_{s1}^{2}/\sigma_{n}^{2})$$

(6) $${\mathrm{SPR}}_{p}=10\log_{10}(\sigma_{s1}^{2}/\sigma_{sp}^{2}),p=1,2,\cdots ,P$$

On the basis of (1)–(4), for each array, we obtain a mapping relationship G: $R^{DP}$→$C^{M}$ (D denotes the dimension of position space; generally, D=2 or 3) from the space of the source position $u={\left[u_{1}^{T},\;u_{2}^{T},\cdots ,\;u_{P}^{T}\right]}^{T}$ to the space of the antenna element output $x_{j}(t)={\left[x_{1j}(t),\;x_{2j}(t),\cdots ,\;x_{M_{j}j}(t)\right]}^{T},j=1,2,\cdots ,J$. By combining information from the J arrays, a new mapping relationship G': $R^{DP}$→$C^{MJ}$ can be obtained from the space of the source position $u={\left[u_{1}^{T},\;u_{2}^{T},\cdots ,\;u_{P}^{T}\right]}^{T}$ to the space of all antenna element outputs $x(t)={\left[x_{1}^{T}(t),\;x_{2}^{T}(t),\cdots ,\;x_{J}^{T}(t)\right]}^{T}$.

## 3. A Modular NN for DPD

**Figure 1:**
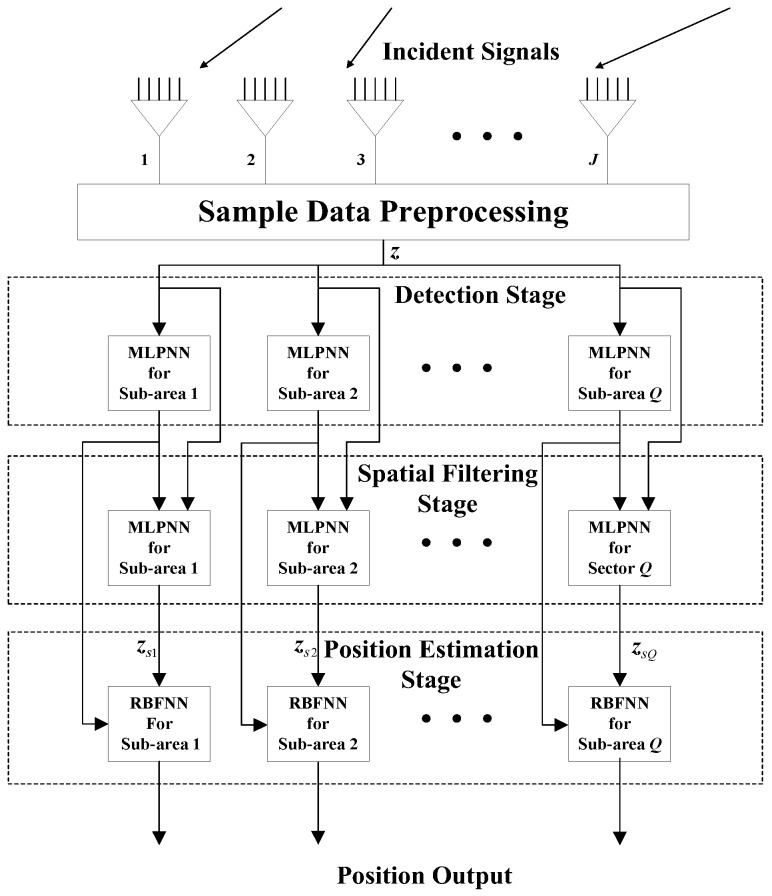
The block diagram of the MLP-MLP-RBF network.

In this section, we propose a modular NN (Figure 1) combining two MLP-NN modules and one RBF-NN module for DPD. This MLP-MLP-RBF network consists of four parts: sample data preprocessing, detection, spatial filtering, and position estimation. In the sample data preprocessing phase, we calculate the covariance matrix ${\hat{R}}_{j},j=1,2,\cdots ,J$ and perform a dimension reduction for each observer. A joint dimension-reduced structure z (Section 3.1) is input into the detection and spatial filtering stage. We choose the covariance matrix as the NN input because it contains enough statistical information about the position of the signal. The area of interest is divided into a number of sub-areas. For each specific sub-area, one MLP-NN is trained to detect the presence of a signal, one MLP-NN is trained to filter out signals outside that sub-area, and another RBF-NN is trained to produce an estimation of the source position. When the NN in the detection stage detects a source in a certain sub-area, the NNs in the corresponding spatial filtering stage and the position estimation stage are activated to complete the spatial filtering and the target position estimation.

This subdivision of region can greatly reduce the size of the training set. Assuming that there are Y training samples in a single source scenario, the number of training samples increases to $Y^{P}$ in the presence of P sources. If subdivision of region is not implemented, the size of the training set is enormous, because it grows exponentially with the number of sources. However, there is also a trade-off between memory resources and positioning accuracy. A smaller sub-area will result in a greater number of NNs, but the accuracy will be improved. In practice, the number of NNs for the proposed MLP-MLP-RBF method depends on the available memory resources.

In addition to the huge reduction for the size of the training set, there are two reasons why we use multistage processing:

(1) For multiple-source localization scenarios, a single-stage network has the problem that the same input data corresponds to different outputs, which leads to the failure of convergence in the network training process. For example, we consider the case of two sources located at $u_{1}=(x_{1},y_{1})$ and $u_{2}=(x_{2},y_{2})$. The output vectors ${\left[x_{1},y_{1},x_{2},y_{2}\right]}^{T}$ and ${\left[x_{2},y_{2},x_{1},y_{1}\right]}^{T}$ correspond to the same input data, which will result in the confusion and make the network unable to converge in the training process, and this situation gets worse as the number of sources increases. This paper overcomes this problem using the subdivision of region and spatial filtering.
(2) The NN with a single stage requires to know exactly the number of sources to determine the number of network output units, otherwise the localization will fail. In our study, this assumption is not necessary due to the existence of the detection stage.

It is the ingenious combination of multiple stages (detection, filtering, and position estimation) that enables NN-based multiple-source DPD. Moreover, the detection stage is only responsible for the target detection, which will not affect the final localization performance. The filtering stage may cause performance loss, but the loss is insignificant with effective training. As can be seen from simulation results in Section 4, performance of the proposed method is generally better than that of the MUSIC-DPD algorithm at low SNRs and can approach the ML accuracy at high SNRs.

NN training takes a long time; however, the characteristics of offline and parallel learning mean that real-time positioning will not be affected. Compared to the traditional MUSIC-based DPD algorithm, the proposed method avoids the need for eigenvalue decomposition and peak search processes, which have high computational complexity. The proposed algorithm only needs to complete a series of addition and multiplication operations to estimate the source position, a procedure that is more suitable for real-time applications. In addition, experimental results show that the positioning accuracy of the MLP-MLP-RBF is comparable to that of the traditional MUSIC-based DPD algorithm.

To reduce the size of the dataset required for the training process, the MLP-NNs in the detection stage are trained with fewer sample data as they only classify the input vectors. More refined data are used to train RBF-NNs in the position estimation stage, as these achieve the high-resolution DPD.

**Remark** **1.** *In practical applications, the sources may be located anywhere in space, but generally we can obtain a priori knowledge about the approximate area where the sources are located before localization. We can subdivide the corresponding region based on this prior knowledge, thus avoiding the subdivision of the whole space. Even if this prior knowledge cannot be obtained, we can use an AOA-based two-step processing estimator [23], including AOA estimation using the MUSIC algorithm and the pseudo-linear weighted least-square (PLWLS) location method using the AOA estimates, to obtain the initial estimate of the source positions, and then subdivide the corresponding region according to this initial estimate.*

**Remark** **2.** *If a source is located in the frontier of a sub-area, false or missed detections for this source may occur, which may result in the failure of the final localization, although it is unlikely to happen. To overcome this problem, we can adjust the centers of sub-areas when the output of a certain NN in the detection stage is around 0.5.*

**Remark** **3.** *The resolution capability of the proposed technique is affected by the size of the sub-area. In practice, we can improve the resolution of the proposed method by further subdividing the sub-areas, of course at the cost of more memory resources. This phenomenon also reflects a trade-off between memory resources and resolution capability.*

### 3.1. Sample Data Preprocessing

In the sample data preprocessing phase, we conduct the following tasks: (1) eliminate the dependency of the covariance matrix on SNR; (2) eliminate the dependency of the covariance matrix on $\sigma_{s1}^{2}$ by dimension reduction and normalization. We first compute the covariance matrix for each array and denote the elements of this matrix by

(7) $$R_{j}=\left[\begin{array}{cccc} R_{11}^{j} & R_{12}^{j} & \cdots & R_{1M}^{j}\\ R_{21}^{j} & R_{22}^{j} & \cdots & R_{2M}^{j}\\ \vdots & \vdots & & \vdots \\ R_{M1}^{j} & R_{M2}^{j} & \cdots & R_{MM}^{j} \end{array}\right],j=1,2,\cdots ,J$$

Exploiting the estimated noise power $\sigma_{n}^{2}$, ${\bar{R}}_{j}=R_{j}-\sigma_{n}^{2}I$ is calculated as

(8) $${\bar{R}}_{j}=\left[\begin{array}{cccc} R_{11}^{j}-\sigma_{n}^{2} & R_{12}^{j} & \cdots & R_{1M}^{j}\\ R_{21}^{j} & R_{22}^{j}-\sigma_{n}^{2} & \cdots & R_{2M}^{j}\\ \vdots & \vdots & & \vdots \\ R_{M1}^{j} & R_{M2}^{j} & \cdots & R_{MM}^{j}-\sigma_{n}^{2} \end{array}\right],j=1,2,\cdots ,J$$

Using all of the elements in (6) as input will result in a high-dimensional input space. To reduce the dimension of the training samples without loss of information, we make full use of the Hermitian feature of ${\bar{R}}_{j}^{}$, and can therefore consider only the elements of the upper triangular part of (6). Furthermore, if it is a uniform linear array, then when the sources are uncorrelated, all entries of the covariance matrix starting from the second row are linear combinations of the entries in the first row [9]. Eventually, the first row of the covariance matrix is sufficient to represent the entire covariance matrix. 

Collecting the elements from the first row of the covariance matrix [10] together, we have

(9) $$b_{j}={\left[R_{11}^{j}-\sigma_{n}^{2},R_{12}^{j},\cdots ,R_{1M}^{j}\right]}^{T}$$

As the NN does not deal directly with complex numbers, we need to extract the real and imaginary parts of each element in $b_{j}$ to reconstruct a (2M−1)-dimensional vector:

(10) b¯j=[Re(R11j−σn2),Re(R12j),Im(R12j),⋯,Re(R1Mj),Im(R1Mj)]T

${\bar{b}}_{j}$ is normalized as

(11) $$z_{j}=\frac{{\bar{b}}_{j}}{\left||{\bar{b}}_{j}|\right|}$$

All elements from [13] for all of the arrays are then collected in the (2M−1)J×1 vector:

(12) $$z={\left[z_{1}^{T},z_{2}^{T},\cdots ,z_{J}^{T}\right]}^{T}$$

Next, z is fed into the NN as the input vector.

According to (3), it can easily be obtained that

(13) $${\bar{R}}_{j}=A_{j}R_{S}A_{j}^{H}=\sigma_{s1}^{2}A_{j}\left[\begin{array}{cccc} 1 & 0 & \cdots & 0\\ 0 & \sigma_{s2}^{2}/\sigma_{s1}^{2} & \cdots & 0\\ \vdots & \vdots & \ddots & \vdots \\ 0 & 0 & \ldots & \sigma_{sP}^{2}/\sigma_{s1}^{2} \end{array}\right]A_{j}^{H},j=1,2,\cdots ,J$$

Therefore, we eliminate the dependence of the covariance matrix on the signal power $\sigma_{s1}^{2}$ after normalization.

After our preprocessing scheme, the number of input neurons in the input layer is reduced from $2M^{2}J$ to (2M−1)J. The proposed preprocessing scheme removes a large number of redundant or irrelevant information and reduces the dimension of the signal parameter space. Hence, the network can be trained more effectively.

### 3.2. Detection Stage

The “detection stage” uses MLP-NNs with two hidden layers. MLP-NNs are known to be powerful classifiers that can provide superior performance to other classifiers [24]. The MLP-NNs give a global approximation of a nonlinear mapping, so they may require a long training time. In the particular case, the training convergence speed of this network type is slower, but more accurate than that of the RBF-NN, which is the main reason we apply MLP-NNs in the detection stage. As a classifier, the output of an MLP network is 0 when the sources are outside the observed sub-area and 1 when the source is inside the associated sub-area. The number of neurons in the input and output layers is (2M−1)J and 1, respectively. In addition, we can use fewer training samples because of the binary nature of the output. Note that the “tan-sigmoid” activation functions are used for all neurons in the hidden layer. The output of l-th neuron in the hidden layer can be expressed as

(14) $$f_{l}(z)=\frac{2}{{\left[1+\exp(-2(w_{l}^{T}z+b_{l}))\right]}^{-1}}-1$$

where z represents the input of NN and $w_{l}$ and $b_{l}$ represent the weight vector and the bias, respectively, with respect to the l-th neuron in the hidden layer. The “purelin” activation functions are used for neurons in the output layer.

**Figure 2:**
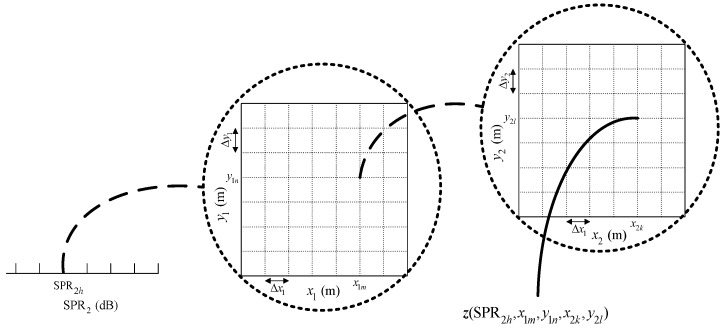
Illustration of the training data structure.

For each MLP-NN in this stage, the specific training steps are as follows (we consider the case of two sub-areas, as illustrated in Figure 2, and do not permit two sources in one sub-area).

- Step 1. Construct the training set for the grid format.
  (1) Predefine a training step (or resolution) denoted by $\Delta x_{1}$, $\Delta y_{1}$, $\Delta x_{2}$, and $\Delta y_{2}$, and calculate $z({\mathrm{SPR}}_{2h},x_{1m},y_{1n},x_{2k},y_{2l})$ using (10).
  (2) Form a training set ${\left\{0(or1)|z^{\left(n\right)}\right\}}_{n=1}^{n=N_{train}}$ of $N_{train}$ observations, in which 1 represents the existence of the source and 0 indicates no signals within the corresponding sub-area. Here, $z^{\left(n\right)}$ is the input in the training process, and 0 (or 1) is the expected output, i.e., the supervision signal.
- Step 2. Construct and train the MLP-NNs properly.
  (1) Construct the appropriate network structure (specific construction process is described in Section 4.1).
  (2) Feed the samples from the training set into the MLP-NN successively, and use Bayesian regularization (BR) [25] to train the NN (the BR algorithm is widely used because of its excellent generalization performance and prevention of overfitting).
- Step 3. Generate a validation set to test the generalization performance of the trained network. The verification set is generated in a similar way to Step 1, but with an offset Δshift based on the training step in Step 1.

**Remark** **4.** *In Step 1, it is not necessary to set variations of the SNR in the training set, because the input to the NNs (the vector z) eliminates the dependency on the SNR.*

### 3.3. Spatial Filtering Stage

The purpose of the spatial filtering stage is to eliminate signals from outside the corresponding sub-area. The MLP-NNs are still used, but unlike in Section 3.2, they now act as filters. The number of neurons in both the input and output layers is (2M−1)J. When the output of the NN in the detection stage is 1, the NN of the corresponding sub-area in this stage is activated.

**Figure 3:**
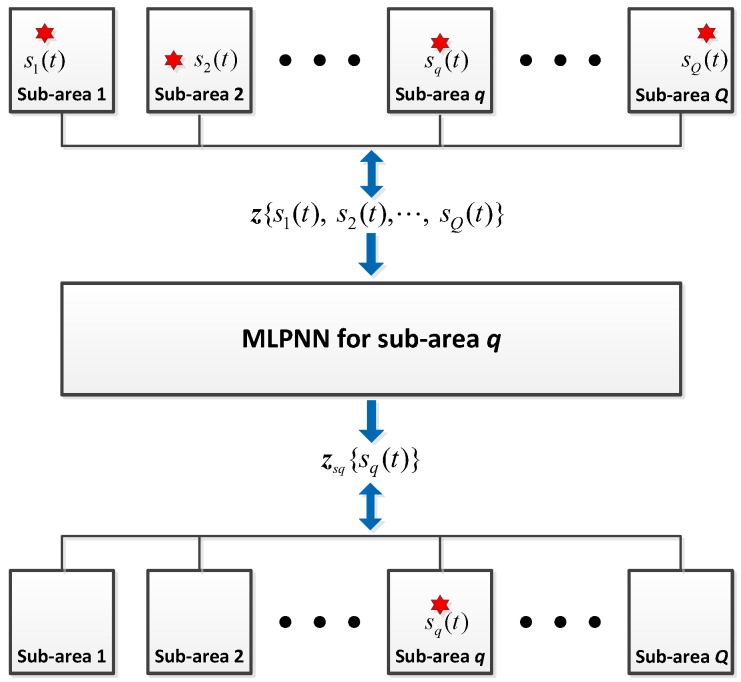
Illustration of the spatial filter network.

The inputs to the spatial filtering network are the vectors z, as for the detection stage. The output of the spatial filter network for sub-area q is $z_{sq},q=1,\cdots ,Q$, which does not include the signals from other sub-areas. For instance, there are Q incident sources $\left\{s_{1}(t),\;s_{2}(t),\cdots ,\;s_{Q}(t)\right\}$, and only source $s_{q}(t)$ is in sub-area q. This procedure can be illustrated in Figure 3, where the red star represents the source. The input to the NN, z, is computed through Equation (10) using signals $\left\{s_{1}(t),\;s_{2}(t),\cdots ,\;s_{Q}(t)\right\}$, and the output $z_{sq}$ is computed through Equation (10) again, but the signal is now $s_{q}(t)$. Through the training process, the MLP-NNs learn and generalize this mapping $\left(z\to z_{sq}\right)$. In the testing phase, an accurate filtering result can be obtained for samples from outside the training set. The training process is similar to that described in Section 3.2 and is not repeated here.

### 3.4. Position Estimation Stage

The NNs in this stage are trained to estimate the source position. When the output of the NNs in the detection stage is 1, the NN of the corresponding sub-area in this stage is activated. The number of neurons in the input and output layers is (2M−1)J and D (the dimension of position space), respectively.

This stage uses an RBF-NN, which is characterized by a radial basis function as the transmission function for hidden layer neurons. The most commonly used radial basis function is the Gaussian function:

(15) $$\phi_{l}(z_{sp})=\exp(-\left||z_{sp}-\mu_{l}|\right|/2\sigma_{l}^{2})$$

where l represents the l-th hidden layer neuron and $\mu_{l}$ and $\sigma_{l}^{2}$ represent the center and the width of the Gaussian activation function, respectively. A three-layer RBF-NN is a local approximation of a nonlinear mapping and can approximate any nonlinear function. It has a faster learning convergence rate than MLP-NN, which is why we apply RBF-NNs in the position estimation stage.

The parameters that must be trained in the RBF-NN are the appropriate number of hidden layers L, the center and width ${\left\{\mu_{l},\sigma_{l}^{2}\right\}}_{l=1}^{L}$, and the weight between layers W. To select reasonable values for these parameters, the training process is as follows:

- Step 1. Construct a training set ${\left\{u_{p}|z_{sp}^{\left(n\right)}\right\}}_{n=1}^{n=N_{train}}$ as for Step 1 in Section 3.2. The only difference is that because of the existence of the spatial filtering network, the training set for the RBF-NN only needs to be constructed in the corresponding sub-area of responsibility, thus reducing the number of training samples. Here, $z_{sp}^{\left(n\right)}$ obtained from the sample data preprocessing is the input in the training process, and the true source position $u_{p}$ is the expected output, i.e., the supervision signal.
- Step 2. Train the network by combining unsupervised and supervised learning strategies. Let L=:1 and compute the following in turn.
  (1) Adopt unsupervised learning (expectation maximum (EM) algorithm [26]) to initialize some inherent parameters ${\left\{\mu_{j},\sigma_{j}^{2}\right\}}_{j=1}^{J}$ in the hidden layer.
  (2) Feed the training samples generated in Step 1 into the RBF-NN successively, and use a supervised learning strategy (Levenberg–Marquardt (LM) algorithm [27]) to determine the weight W.
  (3) Calculate the root mean square error (RMSE) and the number of neurons in the hidden layer. If one of them reaches the predefined criteria, pause the iteration process; otherwise, set L=:L+1 and return to (1).
- Step 3. Generate a validation set to test the generalization performance of the trained network. The specific process is similar to that of Step 3 in Section 3.2.

In the next section, we illustrate our proposed NN approach via a series of numerical simulations.

## 4. Simulation Results

**Figure 4:**
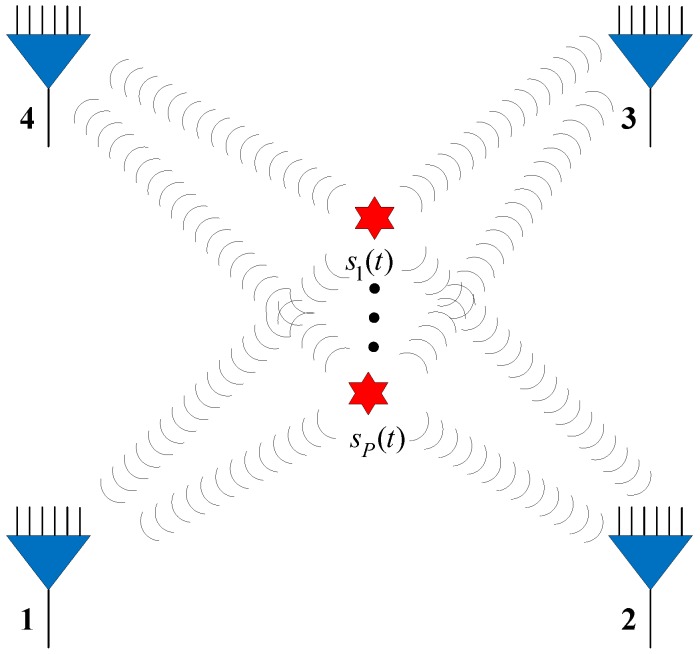
Simulation environment.

In this section, we study the performance of the NNs in the detection, filtering, and position estimation stages. The performance of the proposed network (Section 3) is then evaluated for different SNRs, SPRs, and source location parameters $u_{p}=(x_{p},y_{p}),p=1,\cdots ,P$. In the experiments, we assume there are four available observer arrays, as presented in Figure 4 (the red star and blue triangle represent the source and observer array, respectively), which can receive and locate the radiated source signals. The positions of the observers are listed in Table 2. Each observer array is equipped with a six-element uniform linear array (ULA).

The number of snapshots is set to K=200. To avoid spatial aliasing, the distance between adjacent elements in the ULA was set to half a wavelength, that is, d=λ/2. 

**Figure 5:**
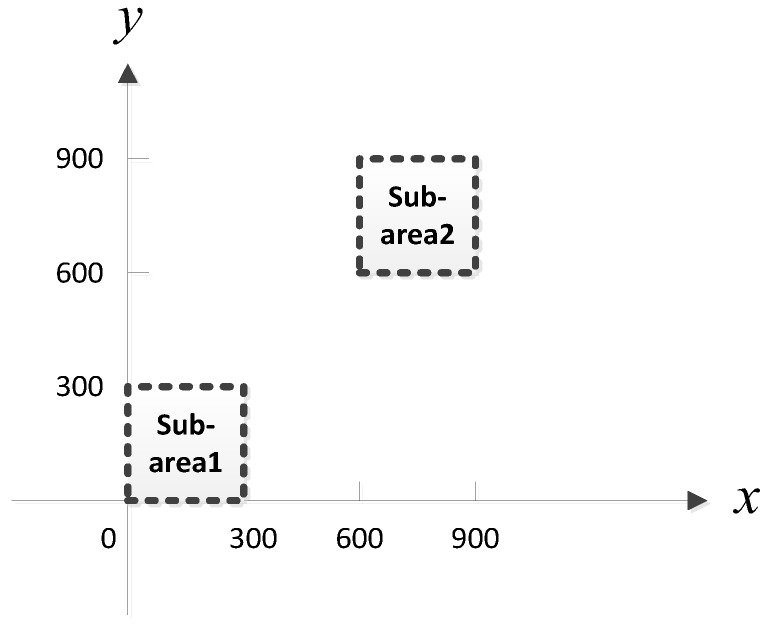
Two sub-areas with 300 m by 300 m.

Considering that MUSIC-based and ML-based methods have better performance than the classical beamforming-based method for small AOA separation, in the simulation, we compare the proposed MLP-MLP-RBF method with MUSIC-based and ML-based DPD methods [14] to show the superiority of our method. To reduce the computational burden of MUSIC-DPD and facilitate the analysis, we study a special case of two sub-areas, as shown in Figure 5. We assume that there is at most one source in each sub-area. In addition, the MUSIC-DPD search strategy uses 20 m coarse search steps and 1 m fine search steps to reduce the computational complexity.

In the training patterns, the training step (or grid resolution) is set to $\Delta x_{1}=\Delta y_{1}=\Delta x_{2}=\Delta y_{2}=20\;(m)$ (Section 3.2, Figure 2) and an offset of Δshift=5 (m) is used for the validation patterns.

**Figure 6:**
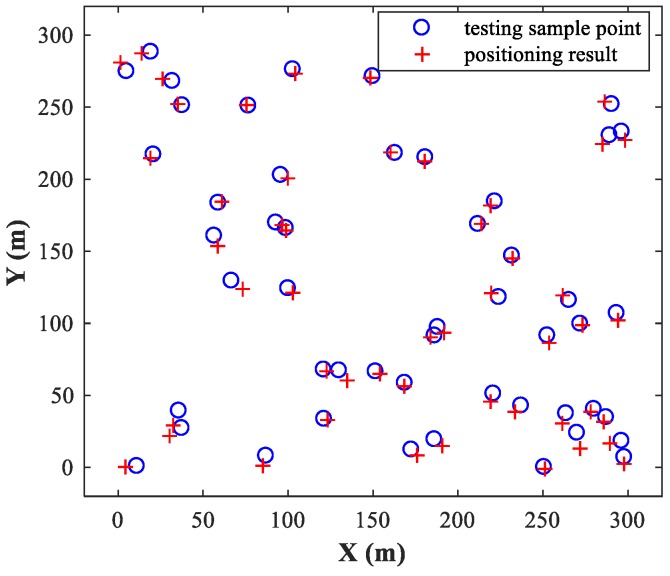
Simulation results of MLP-MLP-RBF for sub-area 1.

**Figure 7:**
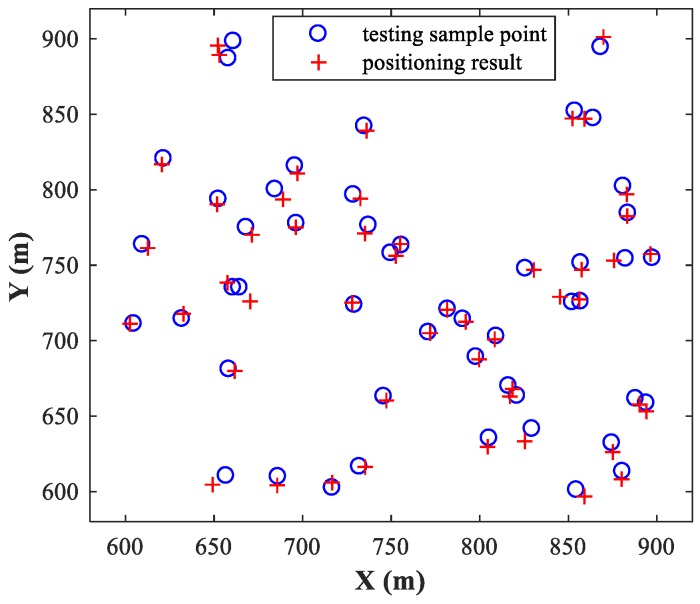
Simulation results of MLP-MLP-RBF for sub-area 2.

The MLP-NN with 12 neurons in both hidden layers is applied in the detection stage, and the MLP-NN with two hidden layers of 24 neurons each is applied in the spatial filtering stage (The reasons for this structural configuration are discussed in Section 4.1). We set SPR=0 dB and SNR=30 dB, and then randomly select 50 pairs of test samples within the two sub-areas and locate them using the trained MLP-MLP-RBF network. Figure 6 and Figure 7 show the simulation results for sub-area 1 and sub-area 2, respectively, where the actual and estimated locations are represented by the circle and crisscross symbols, respectively. The simulation results verify the feasibility of the proposed MLP-MLP-RBF method for DPD and demonstrate the high positioning accuracy of our approach.

In the following subsections, the positioning performance of the proposed MLP-MLP-RBF method is evaluated in detail. We conduct N=500 Monte Carlo (MC) experiments and evaluate the estimation accuracy in terms of the ${\mathrm{RMSE}}_{p}=\sqrt{\frac{1}{N}\sum_{n=1}^{N}||{\hat{u}}_{p}^{n}-u_{p}^{n}|{|}^{2}},p=1,2$, where ${\hat{u}}_{1}^{n}$ and ${\hat{u}}_{2}^{n}$ are the network responses for the n-th MC experiment. Similarly, the mean relative error ${\mathrm{MRE}}_{p}=\frac{1}{N}\sum_{n=1}^{N}\frac{\left||{\hat{z}}_{sp}^{n}-z_{sp}^{n}|\right|}{\left||z_{sp}^{n}|\right|},p=1,2$ is used to evaluate the performance of the filtering network. On this basis, the following figures show boxplots of RMSE or MRE values calculated for different combinations of $\left\{u_{1},u_{2}\right\}$. In other words, in the following simulations, we conduct N=500 MC experiments for each different combination of $\left\{u_{1},u_{2}\right\}$. The boxplots [28] show the minimum, lower quartile, median, upper quartile, and maximum to visually represent the statistical characteristics of the data. The whiskers extending from each end of the box to the maximum and minimum show the extent of the remaining values. The values beyond the ends of the whiskers are treated as outliers. The whisker length is set to 1.5 times the interquartile range. The purpose of applying boxplots in this paper is to illustrate the RMSE and MRE for different combinations of source positions on the same coordinates, which results in a more effective visualization of the positioning performance of the proposed method.

Three basic assumptions are made in the simulations: (1) The array manifold is known exactly; (2) there is at most one source in each sub-area; (3) all radiated signals can be received by each observer.

Note that the proposed method makes no assumptions about the array structure: as long as the array manifold is known, it can be applied directly to the nonuniform array scenario (at this point, $b_{j}$ is composed of the elements of the upper triangular part of (8)). Furthermore, the transmitters must radiate steady-state signals, otherwise the performance of the proposed method will deteriorate.

**Remark** **5.** *The simulation data used in this study is computer-generated. The generated two source signals both follow zero mean circular complex Gaussian distribution with different signal powers* $\sigma_{s1}^{2}$ *and* $\sigma_{s2}^{2}$ *. The signals received at the sensors are contaminated with statistically independent complex Gaussian white noise of power* $\sigma_{n}^{2}$ *. The signals and noise are mutually independent. The received signal power is inversely proportional to the square of the distance between the source and observer array.*

### 4.1. Study of the MLP-NN Performance in the Detection Stage

In this subsection, we mainly study the performance of the MLP-NN in the detection stage. The number of hidden layers and neurons in each hidden layer in the MLP-NN is not usually known beforehand, and the optimum value is generally found through an investigation. Following [29], an iterative process is applied to dynamically adjust the network configuration. Finally, an MLP-NN with good statistical properties is obtained. In the simulations described in this subsection, we use the MLP-NNs with two hidden layers each containing 12 neurons. Moreover, according to the analysis in Section 3.2, the number of neurons in input and output layers is (2M−1)J=44 and 1, respectively. The network is trained for 200 epochs.

**Figure 8:**
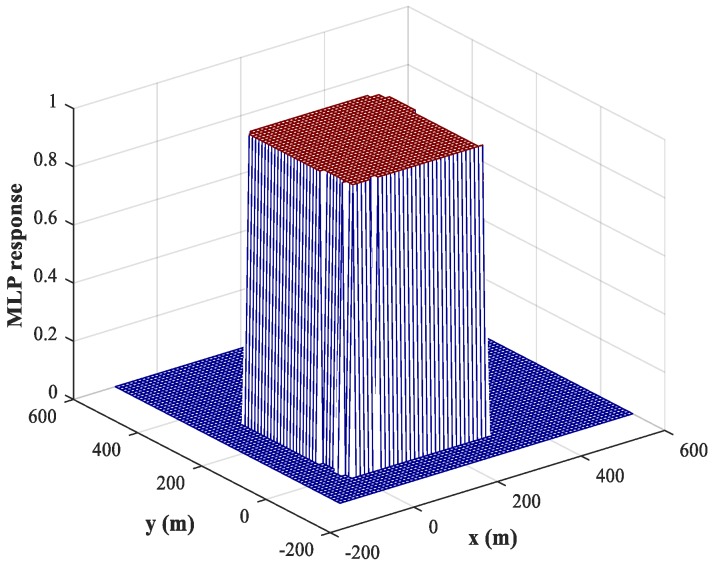
MLP-NN response in the detection stage for sub-area 1 (uniform training step).

**Figure 9:**
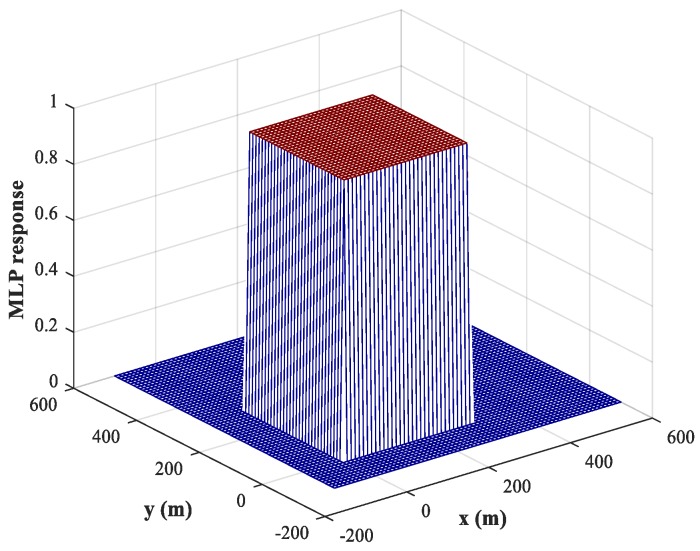
MLP-NN response in the detection stage for sub-area 1 (variable training step).

As mentioned in Section 3.2, we can use a training set containing fewer training samples to train the NNs because of the binary nature of the output. Compared with the spatial filtering and position estimation stages, we set a larger training step, $\Delta x_{1}=\Delta y_{1}=\Delta x_{2}=\Delta y_{2}=50\;(m)$, and hold both SNR and SPR constant. The testing step is set to $\Delta x_{1}=\Delta y_{1}=\Delta x_{2}=\Delta y_{2}=5\;(m)$. The corresponding response of the MLP-NN for sub-area 1 is shown in Figure 8 (similar results are obtained for sub-area 2), from which we can conclude that the trained MLP-NN offers good generalization performance and can also carry out accurate detections for most samples outside the training set. The detection accuracy reaches 98.1%. Moreover, the detection errors tend to occur at the edges of the sub-area. To solve this problem, we train the network using variable training steps. We set a smaller training step of $\Delta x_{1}=\Delta y_{1}=\Delta x_{2}=\Delta y_{2}=10\;m$ within 50 m of the sub-area boundary and keep the training step in other regions constant. The corresponding response of the MLP-NN trained with variable training steps for sub-area 1 is shown in Figure 9, from which we can observe that the detection errors at the edges of the sub-area are significantly reduced and the detection accuracy has increased to 99.8%. Of course, the improvement in detection accuracy is acquired at the cost of computational resources.

**Figure 10:**
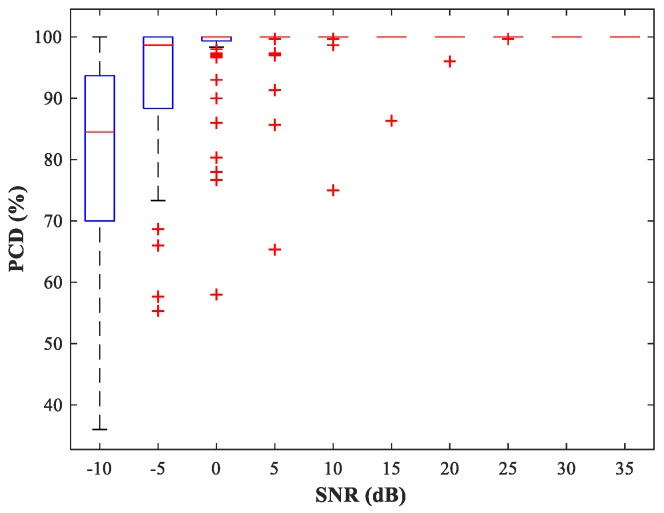
PCD of MLP-NN in the detection stage for sub-area 1 (uniform training step).

**Figure 11:**
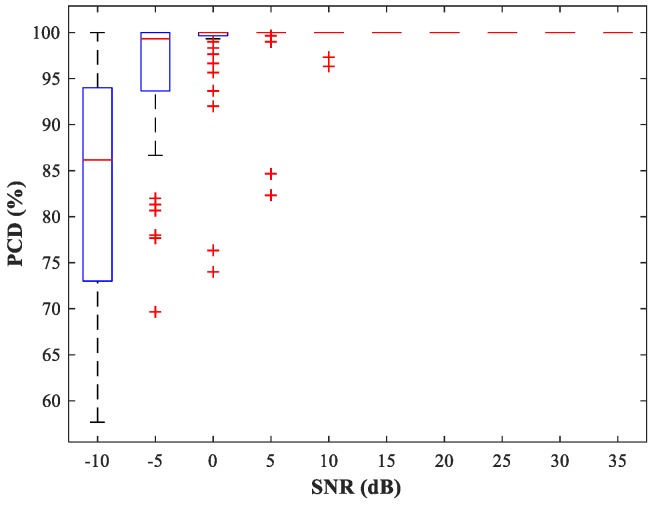
PCD of MLP-NN in the detection stage for sub-area 1 (variable training step).

Next, we fix $u_{2}=(800,800)\;m$ and select the source location $u_{1}$ at random. The performance of the detection network is then evaluated by the probability of correct detection (PCD), which is the ratio of the number of correct detections to the total number of detections. In Figure 10 and Figure 11, we show boxplots of PCD values calculated for 50 different $u_{1}$ and 500 MC experiments under different SNR conditions. Compared with the network trained with uniform steps, the network trained using variable steps has fewer outliers and the detection performance of the latter has improved.

### 4.2. Study of the MLP-NN Performance in the Spatial Filtering Stage

**Figure 12:**
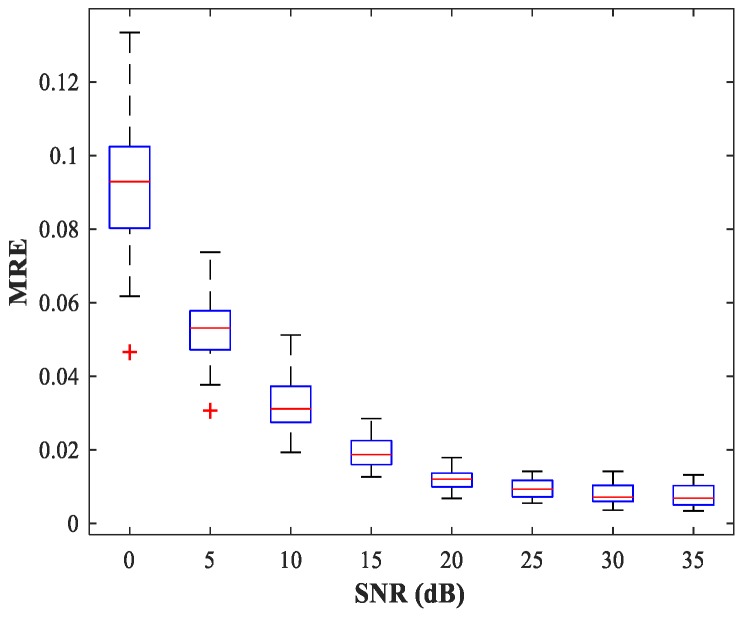
MRE of the MLP-NN in spatial filtering stage versus SNR for sub-area 1. SPR = 0 (dB).

According to the analysis in Section 3.3, the number of input neurons is equal to the number of output neurons in this stage; both are set to (2M−1)J=44 under this simulation scenario. The number of hidden layers and the number of neurons in the hidden layer are determined in the same manner described in Section 4.1. The MLP-NNs with two hidden layers each containing 24 neurons are employed, and the number of neurons in both the input and output layers is (2M−1)J=44. The network is trained for 200 epochs. For constant simulation conditions, Figure 12 shows the curve of MRE versus SNR. The testing set includes 50 random combinations of $\left\{u_{1},u_{2}\right\}$, covering the whole region of the two sub-areas, for SNR values from 0–35 (dB) in steps of 5 (dB). For each combination of SNR, $u_{1}$, and $u_{2}$, we conduct 500 MC experiments.

As can be seen from Figure 12, the trained MLP-NN displays excellent spatial filtering performance, especially under high SNR conditions.

### 4.3. Study of the RBF-NN Performance in the Position Estimation Stage

**Figure 13:**
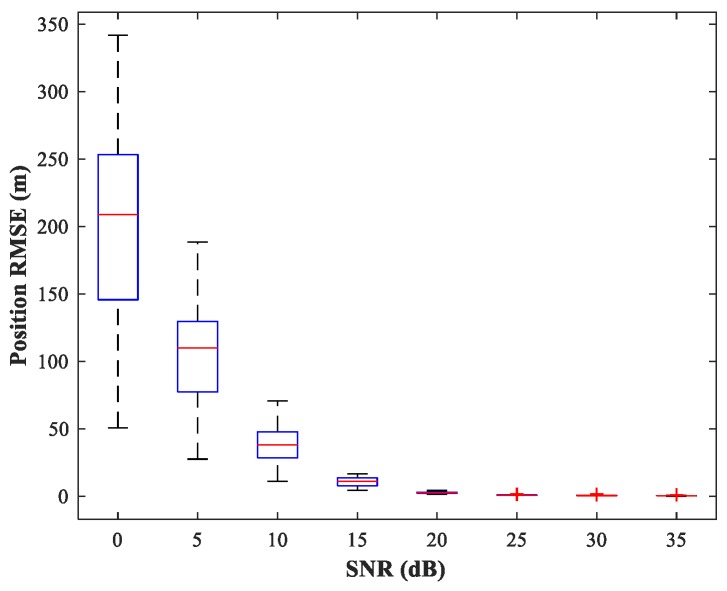
RMSE of the RBF-NN in position estimation stage versus SNR for sub-area 1. SPR = 0 dB.

The existence of a spatial filtering network means that the RBF-NN need only respond to one source in the corresponding sub-area, thus greatly reducing the size of the training set. Following [5], the number of hidden neurons in the RBF-NN is determined by the minimal resource allocation strategy. Finally, the number of neurons in input, hidden, and output layers is (2M−1)J=44, 40, and D=2, respectively. The maximum number of neurons, spread of RBFs, and mean squared error goal are set to 500, 1, and $10^{-5}$, respectively (For the specific training process, see Section 3.4). We consider the same simulation conditions as described in Section 4.2. The simulation results are shown in Figure 13. We can see that the trained RBF-NN offers high localization accuracy and small variance in the high SNR situation (SNR≥15 dB).

### 4.4. Study of the Network Generalization for Different SNRs

**Figure 14:**
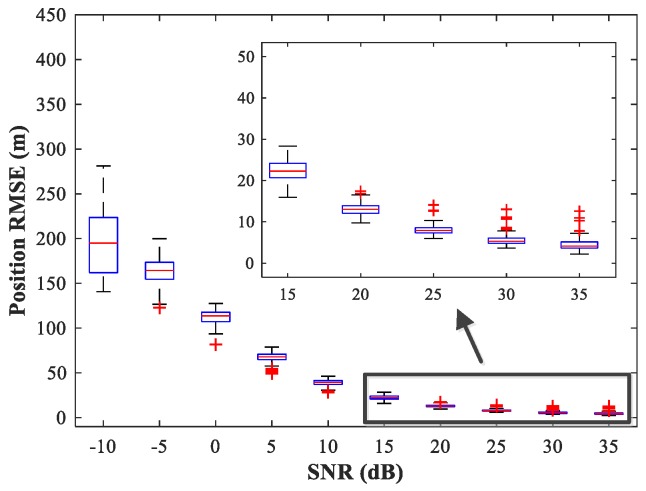
RMSE of the proposed MLP-MLP-RBF network versus SNR for sub-area 1.

**Figure 15:**
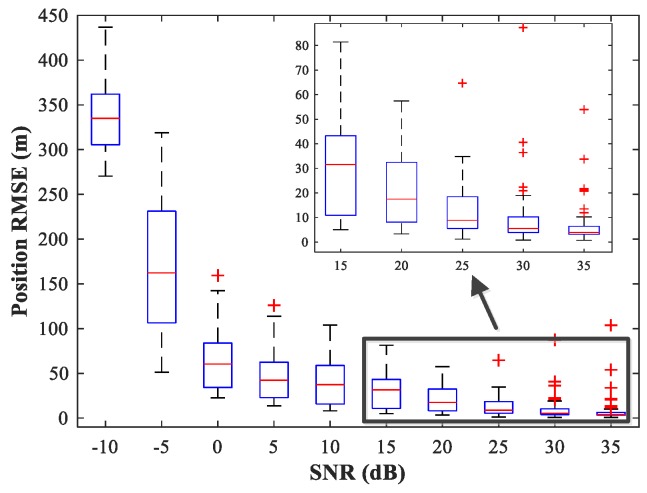
RMSE of the MUSIC-DPD algorithm versus SNR for sub-area 1.

**Figure 16:**
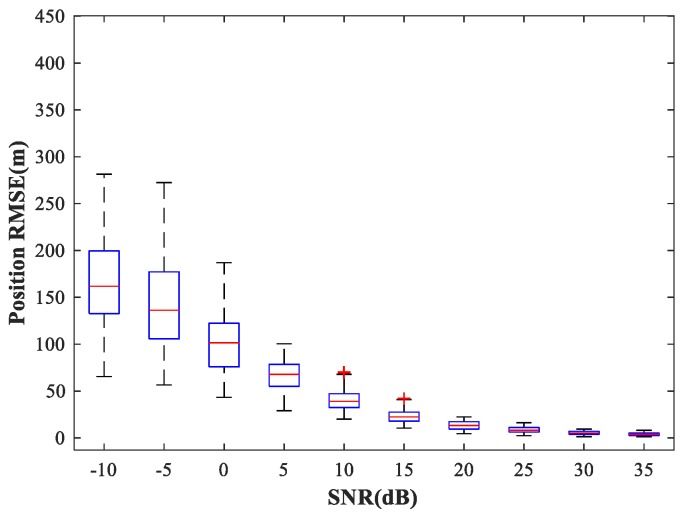
RMSE of the ML-DPD algorithm versus SNR for sub-area 1.

In the following subsections, we combine the previously trained NNs in the manner of Figure 1 and study the performance of the complete MLP-MLP-RBF network. The test set comprises 50 random combinations of $\left\{u_{1},u_{2}\right\}$, covering the whole region of the two sub-areas. We fix SPR = 0 dB and vary the SNR in the range −10–35 dB in steps of 5 dB. Figure 14, Figure 15 and Figure 16 show ${\mathrm{RMSE}}_{1}$ of the proposed MLP-MLP-RBF method, the MUSIC-DPD algorithm, and the ML-DPD algorithm, respectively.

As expected, compared with the MUSIC-DPD algorithm, the proposed MLP-MLP-RBF method has a lower RMSE value at high SNRs (SNR≥15 dB) and smaller variance. Furthermore, we find that the proposed method exhibits better localization performance than the MUSIC-DPD algorithm in the case of low SNRs (SNR≤10 dB). This can be interpreted as the MUSIC-DPD algorithm suffering from the thresholding effect, whereas the MLP-MLP-RBF method is more robust at high noise levels. On the other hand, the proposed MLP-MLP-RBF method can attain the ML accuracy at high SNRs.

### 4.5. Study of the Network Generalization for Different SPRs

**Figure 17:**
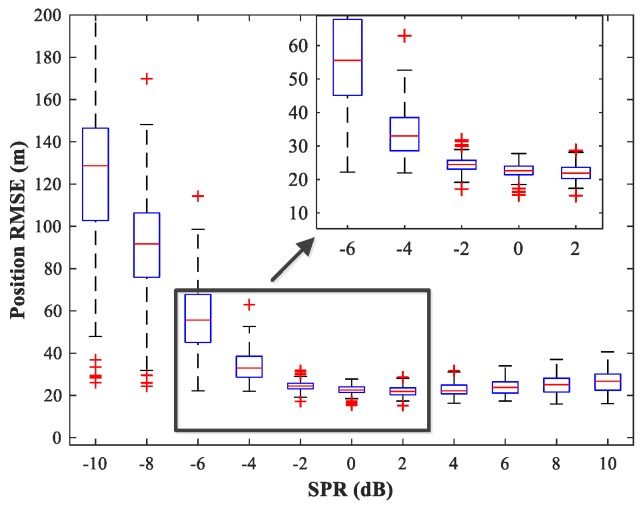
RMSE of the proposed MLP-MLP-RBF network versus SPR for sub-area 1.

**Figure 18:**
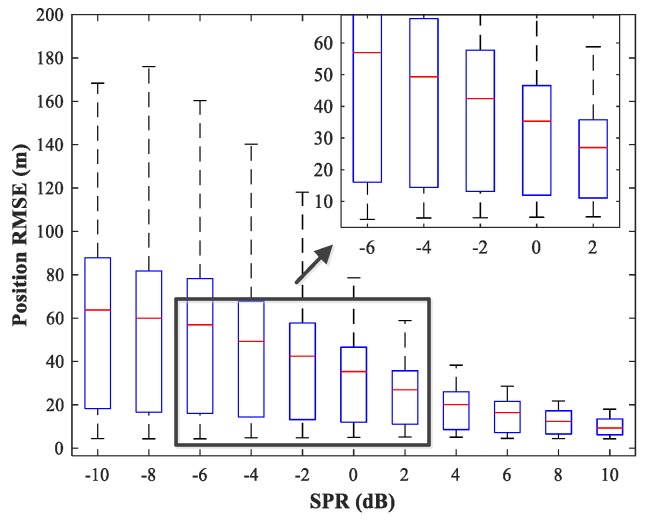
RMSE of the MUSIC-DPD algorithm versus SPR for sub-area 1.

**Figure 19:**
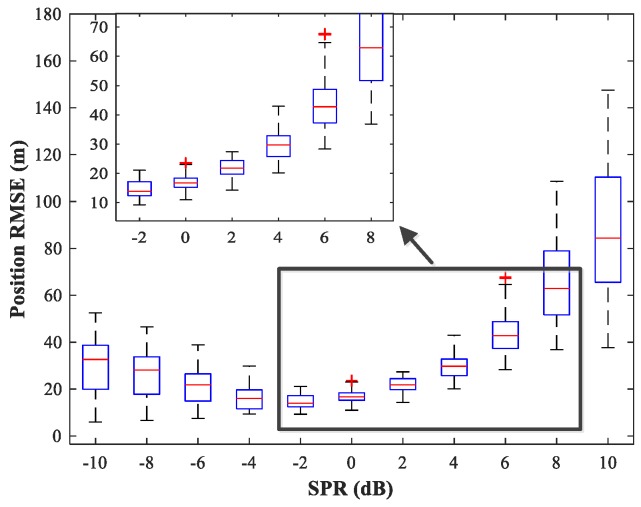
RMSE of the proposed MLP-MLP-RBF network versus SPR for sub-area 2.

**Figure 20:**
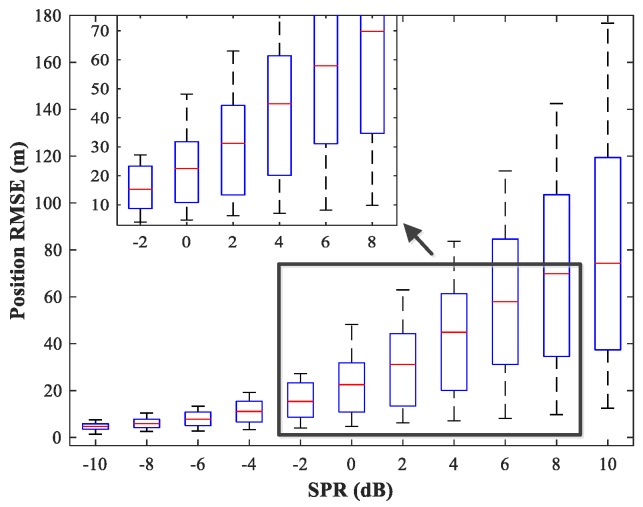
RMSE of the MUSIC-DPD algorithm versus SPR for sub-area 2.

The effect of variations in SPR on the position estimation performance is now studied. The simulation conditions are as previously described. We fix SNR = 15 dB and vary SPR from −10–10 dB in steps of 2 dB. Note that our MLP-MLP-RBF network is trained with SPR = 0 dB, and the goal here is to evaluate the performance of the network with SPR values for which the network is not trained. The simulation results are shown in Figure 17, Figure 18, Figure 19 and Figure 20. As expected, the sources in sub-areas 1 and 2 have high positioning accuracy under high and low SPR conditions, respectively. This can be explained by the fact that with an increase in SPR, the power of the first source becomes higher than that of the second source. From these figures, we can observe that compared with the MUSIC-DPD algorithm, the proposed network offers higher localization accuracy in the range −6≤SPR≤2 dB for sub-area 1 and −2≤SPR≤8 dB for sub-area 2.

From the above simulation results, we can conclude that the proposed MLP-MLP-RBF method performs similarly to the MUSIC-DPD algorithm. The results show that the MLP-MLP-RBF network offers good generalization performance for SNRs and SPRs that do not appear in the training set.

### 4.6. Study of the Network Performance for Different Snapshots

**Figure 21:**
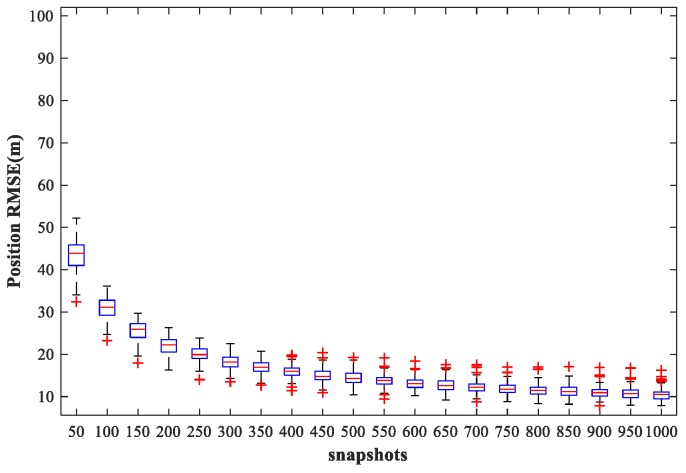
RMSE of the proposed MLP-MLP-RBF network versus the number of snapshots for sub-area 1.

**Figure 22:**
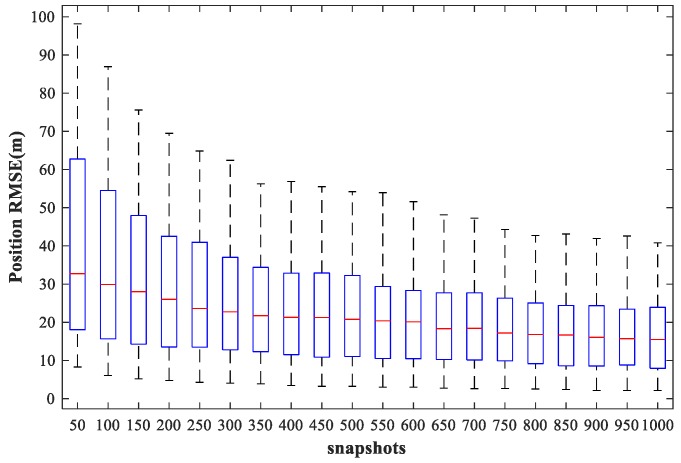
RMSE of the MUSIC-DPD algorithm versus the number of snapshots for sub-area 1.

In this subsection, we examine the localization performance of the proposed method for different snapshots. We fix SPR = 0 dB, SNR = 30 dB, and vary the number of snapshots from 50 to 1000 times in steps of 50 times. Other simulation conditions are as described above. Figure 21 and Figure 22 show the ${\mathrm{RMSE}}_{1}$ of the proposed MLP-MLP-RBF method and the MUSIC-DPD algorithm, respectively, with respect to the number of snapshots.

From the simulation results, we can see that the localization accuracy of both the proposed method and the MUSIC-DPD algorithm increases with the number of snapshots. However, as the number of snapshots continues to increase, the improvement in accuracy slows considerably. Moreover, under these simulation conditions, 200 snapshots are sufficient to meet the required localization accuracy; this is why 200 snapshots were used in the previous simulations.

### 4.7. Comparison of the Processing Time

To highlight the superiority of our MLP-MLP-RBF network, we now compare the processing time of the proposed method with that of the MUSIC-DPD and ML-DPD algorithms. Note that the MUSIC-DPD algorithm uses a search strategy with a 20 m coarse search step and 1 m fine search step. The computations are executed in Matlab R2017a on a PC with Intel Core i7-3520 CPU.

The simulation conditions are set to $u_{1}=(150,220)\;m$, $u_{2}=(830,710)\;m$, SNR=10 dB, and SPR=0 dB, and all other conditions are the same as previously described. The simulations are based on 500 MC runs. The average processing times in each stage and the total runtimes of the two methods are shown in Table 3. Our MLP-MLP-RBF network requires only about 0.04 s to perform position estimation, which is much faster than the MUSIC-DPD and ML-DPD algorithms. The simulation results also indicate that the proposed MLP-MLP-RBF method can effectively solve the real-time multisource localization problem.

### 4.8. Study of the Network Performance for Nonuniform Arrays

**Figure 23:**
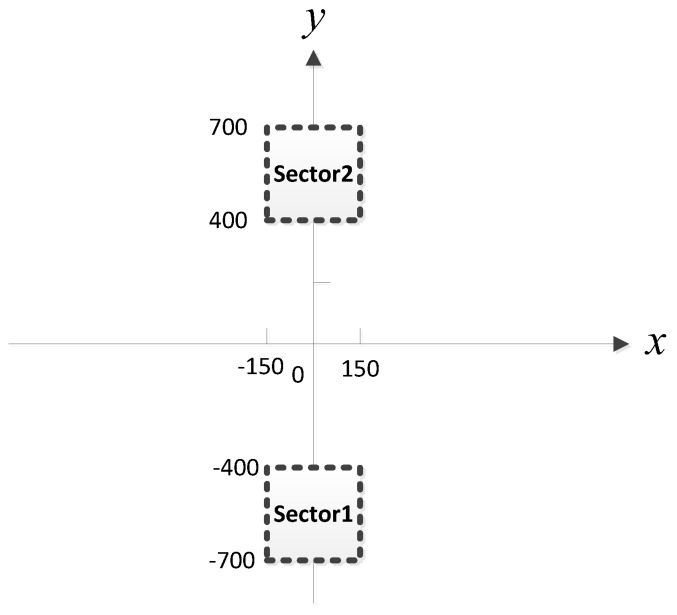
Two sub-areas with 300 m by 300 m.

The purpose of this subsection is to verify the feasibility of the proposed method for nonuniform arrays. The positions of the observers are listed in Table 4. Each observer array is equipped with a six-element nonuniform linear array. For simplicity, the distance between adjacent elements in each array was set to 0.5λ, λ, λ, 1.5λ, and 0.5λ, respectively. Assume that two sources are located in the two sub-areas of Figure 23. The other simulation conditions and the network configuration are as described above.

**Figure 24:**
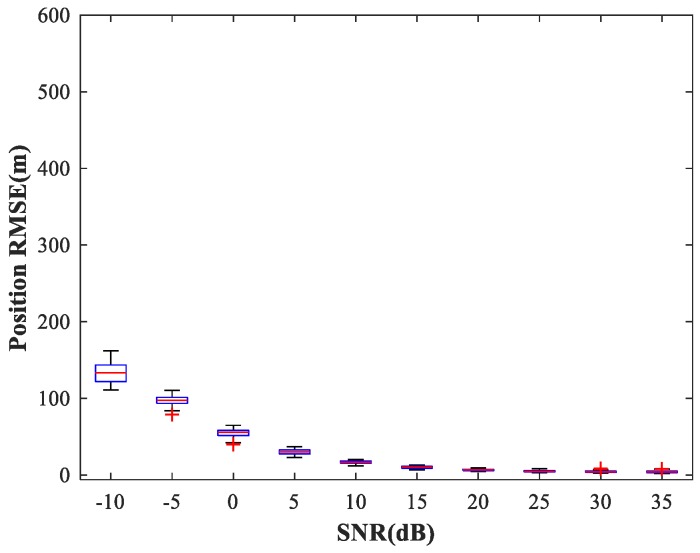
RMSE of the proposed MLP-MLP-RBF network versus SNR for sub-area 1.

**Figure 25:**
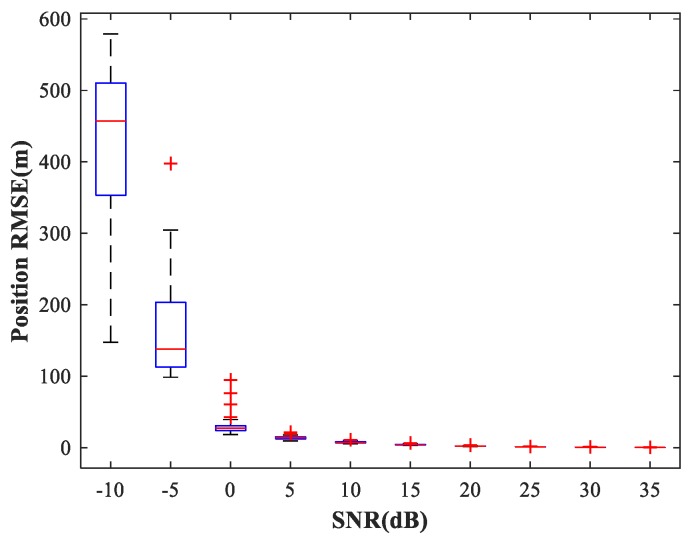
RMSE of the MUSIC-DPD algorithm versus SNR for sub-area 1.

**Figure 26:**
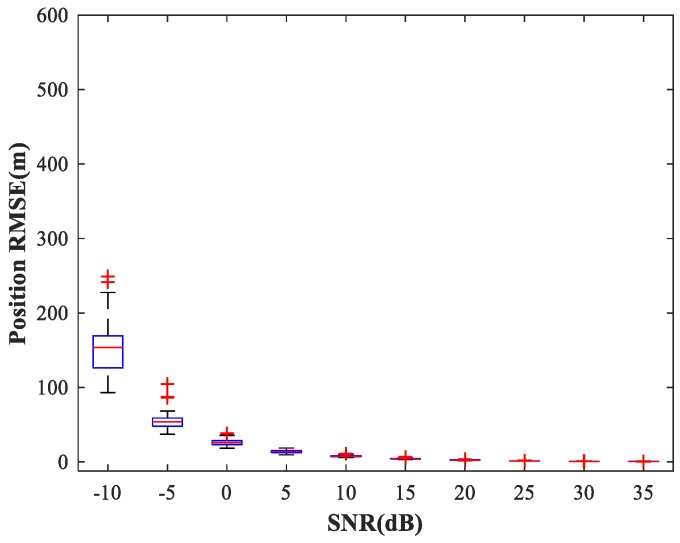
RMSE of the ML-DPD algorithm versus SNR for sub-area 1.

The test set comprises 50 random combinations of $\left\{u_{1},u_{2}\right\}$, covering the whole region of the two sub-areas. We fix SPR = 0 dB and vary the SNR in the range −10–35 dB in steps of 5 dB. The simulation results given by the three algorithms are shown in Figure 24, Figure 25 and Figure 26. As expected, the proposed MLP-MLP-RBF method can be applied to nonuniform arrays directly. Moreover, we can obtain a conclusion similar to that for the uniform array scenario; that is, the proposed method has a higher localization accuracy than the MUSIC-DPD algorithm at low SNRs (SNR<B) and can attain the ML accuracy at high SNRs.

### 4.9. Study of the Network Performance for Different Sizes of Sub-Area

**Figure 27:**
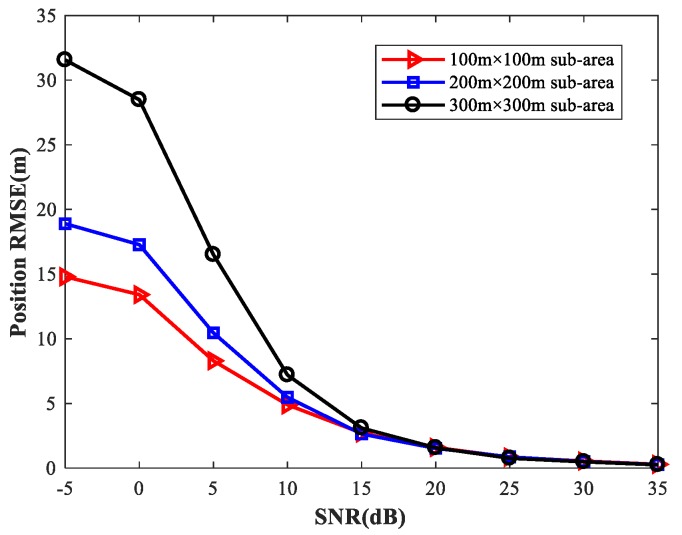
RMSE of the proposed method using four six-element-ULA-equipped observers for different sizes of sub-area.

**Figure 28:**
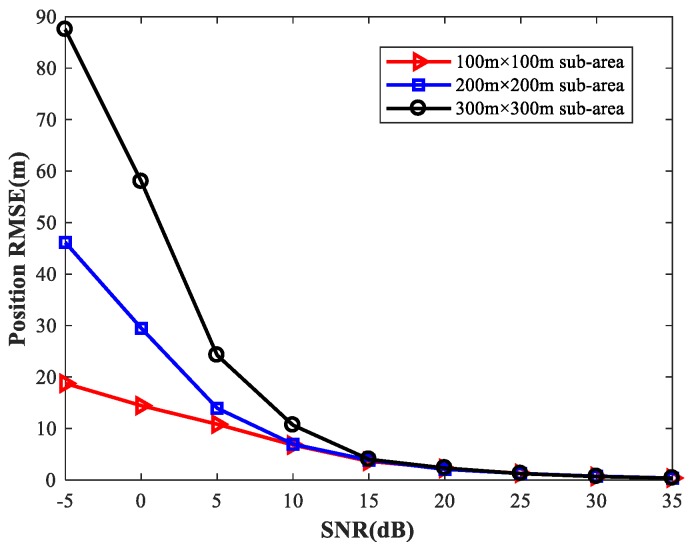
RMSE of the proposed method using two six-element-ULA-equipped observers for different sizes of sub-area.

**Figure 29:**
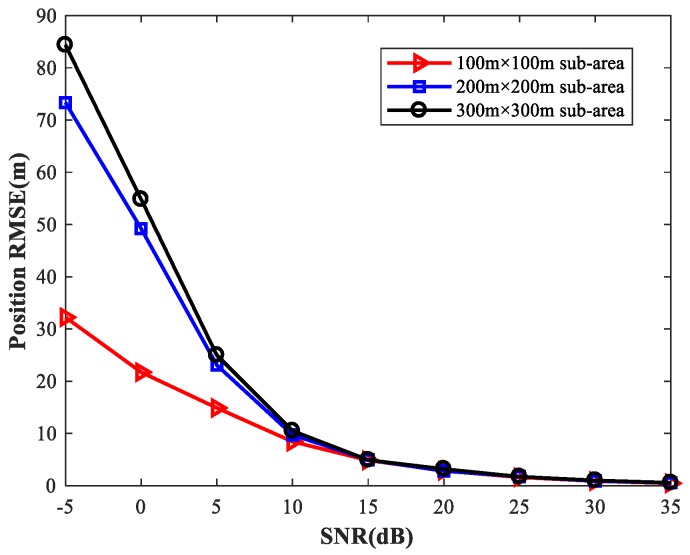
RMSE of the proposed method using four four-element-ULA-equipped observers for different sizes of sub-area.

To provide a general guideline for how to balance memory resources with positioning accuracy, we study the localization performance of the proposed method for different sizes of sub-area with various numbers of observers and antennas. We first set the simulation conditions as previously described. We consider four observers at the positions listed in Table 2. Each observer array is equipped with a six-element uniform linear array. We fix SPR = 0 (dB), $u_{1}=(0,0)\;m$, and select the source location $u_{2}$ at random. Figure 27 shows the ${\mathrm{RMSE}}_{1}$ of the proposed MLP-MLP-RBF method with respect to SNR for different sizes of sub-area (100 m × 100 m, 200 m × 200 m, and 300 m × 300 m). Next, we consider two observers with six antennas and four observers with four antennas, respectively, and the other simulation conditions are as described above. The simulation results are shown in Figure 28 and Figure 29.

From these figures, we can see that a smaller sub-area will result in a higher localization accuracy. Moreover, these simulation results also indicate that the positioning accuracy improves with the number of observers and antennas. Based on this, when there are enough observers and antennas available (the reachable positioning accuracy is high), we can reduce the number of sub-areas and achieve a certain positioning accuracy with less computational resources. If the observers and antennas are relatively scarce, we need to further subdivide the sub-areas to achieve the desired positioning accuracy at the cost of more memory resources.

### 4.10. Comparison of the Resolution Capability for the Proposed Method and MUSIC-DPD Algorithm

**Figure 30:**
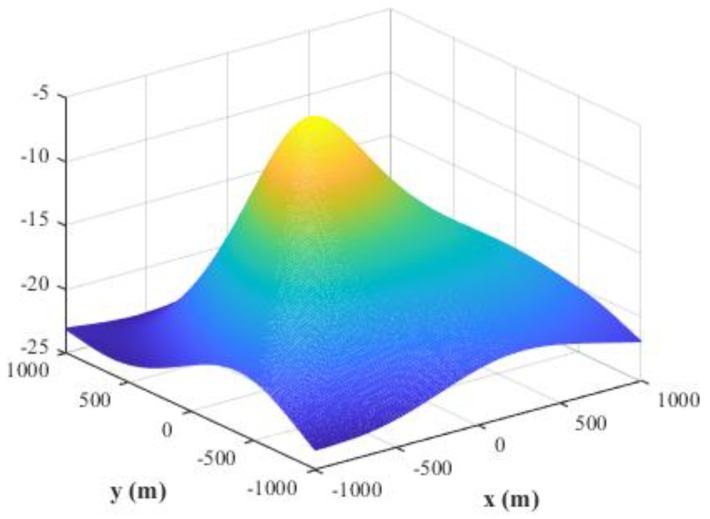
Spectrum of the MUSIC-DPD algorithm.

**Figure 31:**
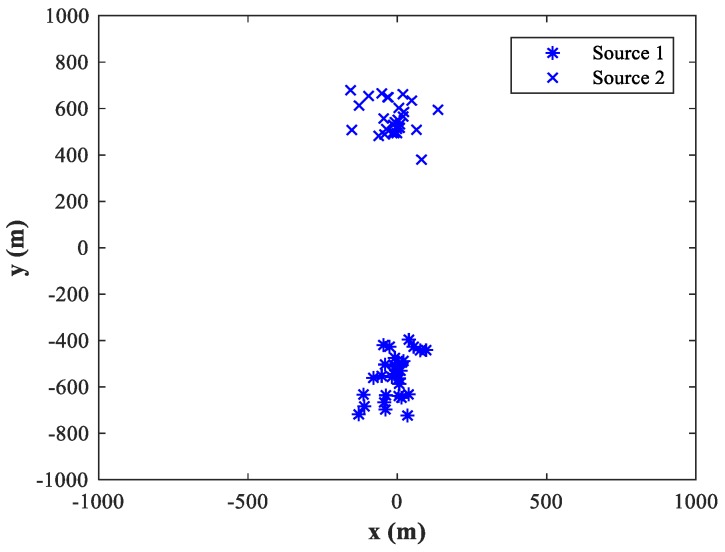
Simulation results of the proposed method for 30 MC experiments.

The main advantage of MUSIC algorithm is its high resolution. In this subsection, we attempt to compare the resolution capability of the proposed MLP-MLP-RBF network with that of MUSIC-DPD method. Since the proposed technique is based on NN computation, it has no spectrum; so, it is difficult to compare their resolution capability under the standard definition of resolution. Next, we design and conduct a set of simulation experiments to reveal that the proposed method has better resolution capability than MUSIC under low SNR conditions. We fix SPR = 0 dB, SNR = −10 dB, $u_{1}=(-500,0)\;m$, and $u_{2}=(500,0)\;m$, and the other simulation conditions are the same as described in Section 4.8. Figure 30 and Figure 31 show the spectrum of the MUSIC-DPD algorithm and simulation results of the proposed method for 30 MC experiments, respectively.

From Figure 30 and Figure 31, we can conclude that when MUSIC-DPD algorithm fails due to the overlap of two original spectral peaks at a low SNR, the proposed MLP-MLP-RBF network can distinguish two sources clearly. The simulation shows the resolution capability of the proposed technique from a certain aspect. As can be seen from Remark 3, the resolution capability of the proposed method depends on the size of the sub-area; however, in practice, we can improve the resolution by further subdividing the sub-areas.

### 4.11. Study of the Network Performance for Other Arrangement of Observer Positions

**Figure 32:**
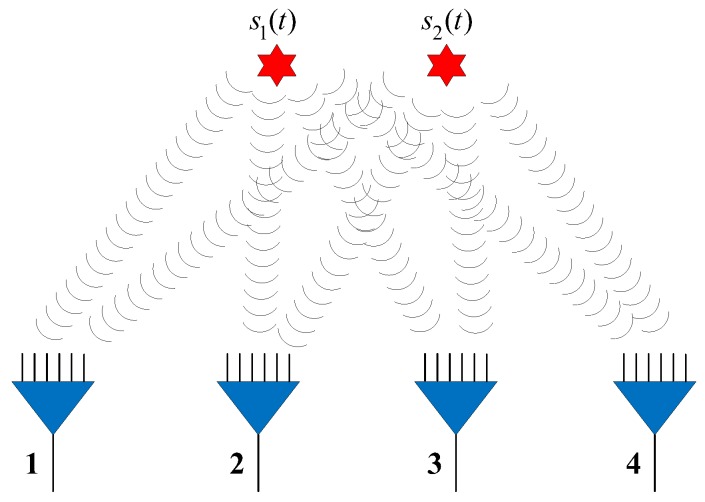
Simulation environment.

**Figure 33:**
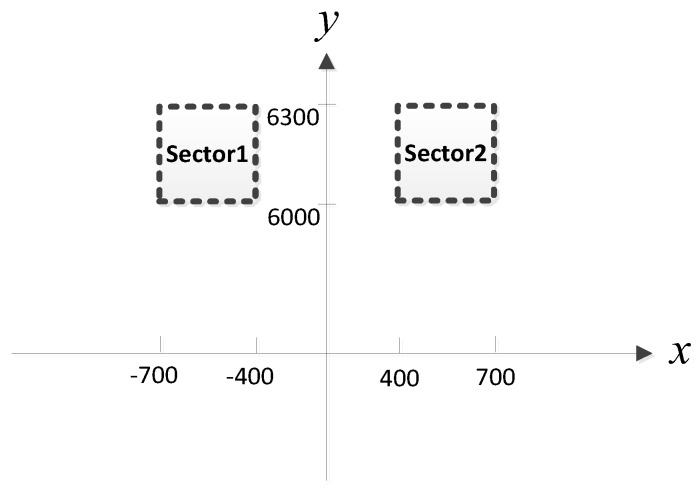
Two sub-areas with 300 m by 300 m.

In this subsection, we examine the localization performance of the proposed method for other observer position arrangement. We consider four observers at the positions listed in Table 5. Without loss of generality, each observer array is equipped with a six-element nonuniform linear array. The simulation environment is shown in Figure 32. Assume that two sources are located in the two sub-areas of Figure 33. The other simulation conditions are the same as described in Section 4.8.

**Figure 34:**
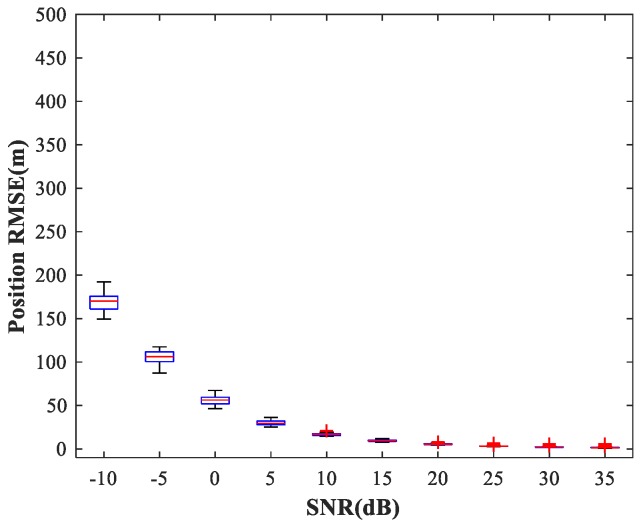
RMSE of the proposed MLP-MLP-RBF network versus SNR for sub-area 1.

**Figure 35:**
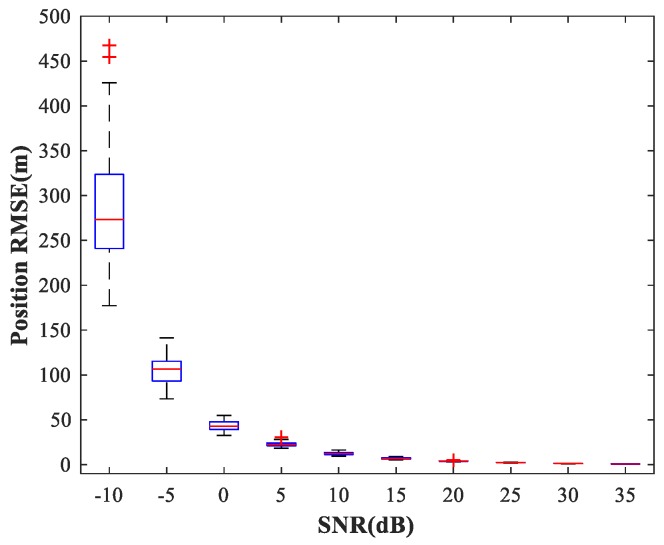
RMSE of the MUSIC-DPD algorithm versus SNR for sub-area 1.

**Figure 36:**
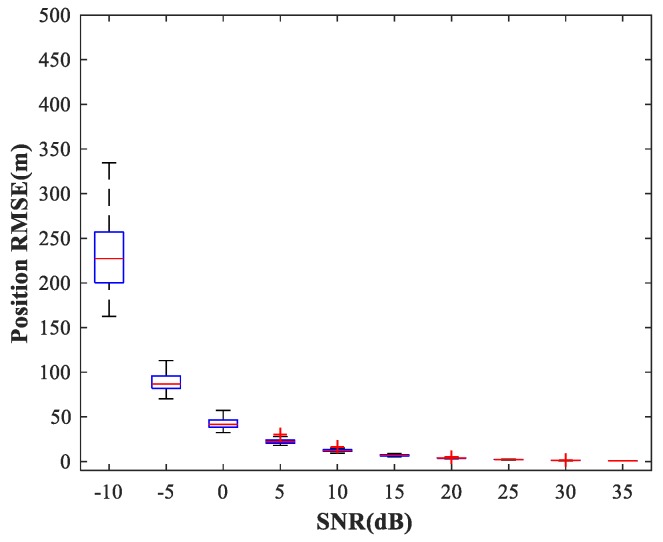
RMSE of the ML-DPD algorithm versus SNR for sub-area 1.

We fix SPR = 0 dB and vary the SNR in the range −10–35 dB in steps of 5 dB. The simulation results for three algorithms mentioned above are shown in Figure 34, Figure 35 and Figure 36. As expected, the proposed MLP-MLP-RBF method can be well-generalized to other observer position arrangements. Furthermore, we can get the same conclusion as that in Section 4.4 and Section 4.8, thus it is not repeated here.

## 5. Conclusions and Future Work

In this paper, we have proposed a new NN-based DPD algorithm (MLP–MLP–RBF) for multiple sources. The MLP-MLP-RBF network performs the DPD through data preprocessing, detection, spatial filtering, and position estimation stages. The originalities of the proposed MLP-MLP-RBF network are the dimension reduction and normalization for all array sample covariance matrixes, the implementation of the spatial filtering network that effectively filters out the sources beyond the relevant sub-area, and the subdivision of region that greatly reduces the amount of training data required. These novel developments enable multiple-source DPD. We have also elaborated on the construction and training processes of two types of NNs in various stages.

Finally, several simulation experiments are conducted to verify the superiority of the new algorithm and the validity of the theoretical derivation. The performance of the NNs in the detection, filtering, and position estimation stages is verified. The proposed MLP-MLP-RBF network shows good generalization performance for a wide range of SNR and SPR values, and its performance is generally better than that of the MUSIC-DPD algorithm at low SNRs and can attain the ML accuracy at high SNRs. Furthermore, a key advantage of this approach over the MUSIC-DPD and ML-DPD algorithms is its ability to provide the DPD almost instantaneously, making it suitable for real-time implementation.

Currently, the proposed method only uses the angle information of the radiated signals in the two-dimensional positioning scene. In future work, we will extend the method to overcome the following issues:

- Multiple target localization combining angle and delay information.
- Location estimation in three dimensions.
- Extending the detection stage to estimate the number of sources for a sub-area.

## Figures and Tables

**Table 1:** Mathematical notation.

Notation	Explanation
I	identity matrix
||⋅||	the Euclidean norm of a vector
Re(⋅)	the real part of an arithmetical expression
Im(⋅)	the imaginary part of an arithmetical expression
${\left(\cdot \right)}^{T}$	transpose
${\left(\cdot \right)}^{H}$	conjugate transpose

**Table 2:** Positions of observer arrays (units: m).

Observer No.	1	2	3	4
$x_{i}$	−2000	2000	2000	−2000
$y_{i}^{}$	−2000	−2000	2000	2000

**Table 3:** Comparison of three algorithms in processing time.

Method	Stage	CPU Average Processing Times/s	Total Time/s
MLP–MLP–RBF	data preprocessing	0.0013	0.0383
	detection	0.0105	
	spatial filtering	0.0146	
	position estimation	0.0119	
MUSIC-DPD	N/A ^a^	1.2027	1.2027
ML-DPD	N/A	6.3508	6.3508

^a^ N/A means the algorithm is not multi-stage.

**Table 4:** Positions of observer arrays (units: m).

Observer No.	1	2	3	4
$x_{i}$	−3000	3000	3000	−3000
$y_{i}^{}$	−3000	−3000	3000	3000

**Table 5:** Positions of observer arrays (units: m).

Observer No.	1	2	3	4
$x_{i}$	−6000	−2000	2000	6000
$y_{i}^{}$	0	0	0	0
